# Observational study of the coverage rate and survival analysis of COVID-19 vaccination among Indigenous people in Roraima, 2021-2023 Individual and contextual factors and professional qualification completion for obesity management: a cross-sectional study, Brazil, 2021-2022

**DOI:** 10.1590/S2237-96222026v35e20251154.en

**Published:** 2026-07-16

**Authors:** Angélica Ribeiro e Silva, Nathália Luíza Ferreira, Patrícia Pinheiro de Freitas, Mariana Souza Lopes, Aline Cristine Souza Lopes

**Affiliations:** 1 Universidade Federal de Minas Gerais, Departamento de Nutrição, Belo Horizonte, MG, Brazil Universidade Federal de Minas Gerais Departamento de Nutrição Belo Horizonte MG Brazil; 2 Universidade Federal de Lavras, Departamento de Nutrição, Lavras, MG, Brazil Universidade Federal de Lavras Departamento de Nutrição Lavras MG Brazil; 3 Universidade Federal dos Vales do Jequitinhonha e Mucuri, Departamento de Nutrição, Diamantina, MG, Brazil Universidade Federal dos Vales do Jequitinhonha e Mucuri Departamento de Nutrição Diamantina MG Brazil; 4 Universidade Federal da Paraíba, Departamento de Nutrição, João Pessoa, PB, Brazil Universidade Federal da Paraíba Departamento de Nutrição João Pessoa PB Brazil

**Keywords:** Obesity, Education, Continuing, Education, Public Health Professional, Unified Health System, Cross-Sectional Studies, Obesidad, Educación Continua, Educación en Salud Pública Profesional, Sistema Único de Salud, Estudios Transversales

## Abstract

**Objective:**

To identify the individual and contextual factors associated with completion of distance professional qualification for the collective management of obesity in the Brazilian Unified Health System (Sistema Único de Saúde, SUS).

**Methods:**

A cross-sectional study with health professionals and students. The 40-hour course included tutoring and was based on material developed by the Ministry of Health. The individual factors analyzed included sociodemographic and professional data collected at the beginning of the course, and the contextual factors comprised economic and demographic data from the States and the Federal District where participants worked, obtained from institutional websites. Participants who completed all activities were considered to have completed the course. Due to convergence failure of the contextual variables in the multilevel logistic regression, a multivariate logistic regression was performed, including adjusted odds ratios (OR) and 95% confidence intervals (95%CI), adjusted for sex, age, and macro-region of the country.

**Results:**

A total of 3,975 individuals enrolled in the course; 1,620 (40.8%) accessed the virtual platform. Most were women (91.0%) and dietitian (56.7%) and worked in the Southeast macro-region (54.8%) and metropolitan areas (51.0%). After model adjustment, being a dietitian (OR 1.55; 95%CI 1.09; 2.19) and having prior access to printed/digital materials on the topic (OR 1.27; 95%CI 1.04; 1.56) were found to increase the odds of completing the professional qualification.

**Conclusion:**

Completion of the professional qualification was associated with being a dietitian and with prior access to printed/digital materials on the topic, highlighting the importance of professional category and prior exposure to the topic in promoting engagement and course completion.

## Resumen

**Objetivo:**

Identificar los factores individuales y contextuales asociados a la finalización de una capacitación profesional a distancia en el manejo colectivo de la obesidad en el Sistema Único de Salud (Sistema Único de Saúde, SUS).

**Métodos:**

Estudio transversal con profesionales y estudiantes del área de la salud. El curso, con una carga horaria de 40 horas, contó con tutoría y se basó en material del Ministerio de Salud. Los factores individuales analizados incluyeron datos sociodemográficos y profesionales obtenidos al inicio del curso, y los factores contextuales abarcaron datos económicos y demográficos de las Unidades Federativas de actuación, obtenidos en sitios institucionales. Los participantes que completaron todas las actividades fueron considerados finalizadores. Debido a la falta de convergencia de las variables contextuales en la regresión logística multinivel, se realizó una regresión logística multivariada, incluyendo la razón de chances (odds ratio, OR) ajustada y el intervalo de confianza del 95% (IC95%) por sexo, edad y macroregión del país.

**Resultados:**

Se registraron 3.975 inscritos en el curso; 1.620 (40,8%) accedieron a la plataforma virtual. La mayoría eran mujeres (91,0%) y nutricionistas (56,7%), y trabajaban en la macroregión Sudeste del país (54,8%) y en regiones metropolitanas (51,0%). Tras los ajustes del modelo, se observó que ser nutricionista (OR 1,55; IC95% 1,09-2,19) y tener acceso previo a materiales impresos o digitales sobre el tema (OR 1,27; IC95% 1,04-1,56) aumentaban la probabilidad de finalizar la capacitación profesional.

**Conclusión:**

La finalización de la capacitación profesional estuvo asociada a ser nutricionista y al acceso previo a materiales impresos/digitales sobre el tema, lo que evidenció la importancia de la categoría profesional y de sucesivos acercamientos al tema para favorecer el compromiso y la finalización del curso.

**Ethical aspects**

This research respected ethical principles, having obtained the following approval data:

Research Ethics Committee: Universidade Federal de Minas Gerais

Opinion number: 4294291

Approval date: 23/9/2020

Certificate of Submission for Ethical Appraisal: 93992418.0.0000.5149

Informed Consent Form: Obtained from all participants before data collection.

## Introducion

Obesity is one of the three central components of the global syndemic ^1^. In 2022, it was estimated that more than 800 million people worldwide had obesity ^2^. In Brazil, its prevalence increased by 13% over 17 years ^3^, and, if current trends continue, it may affect 83 million adults by 2044 ^4^. This scenario raises concerns both because of the potential health impacts of obesity itself and the associated comorbidities ^5^.

Obesity has a multifactorial etiology, which makes its management complex and challenging ^1^. Although the individual approach is widely used, the collective approach constitutes an important strategy for reducing body weight and achieving other benefits, especially when integrated with the individual approach ^6^. Group interventions promote social support and the sharing of experiences, which fosters greater continuity of treatment and potentially greater effectiveness in maintaining weight loss over the long term, compared with the individual approach ^6^. The collective approach represents an important gain in the public health context, as it simultaneously reaches more individuals ^7^.

Content on the collective approach to obesity management remains scarce in professional qualification courses in the health field and in the curricula of health-related courses in Brazil. As a consequence, health professionals working in the Brazilian Unified Health System (Sistema Único de Saúde, SUS) report the absence of or insufficient access to professional qualification courses and instructional materials as barriers to group development ^8^. To address this scenario, it is essential that professionals receive ongoing qualification through courses based on current scientific evidence and aligned with the guidelines and needs of the SUS, as recommended by the National Policy on Permanent Education in Health (Política Nacional de Educação Permanente em Saúde, PNEPS) ^9^.

Considering Brazil’s territorial extent and the complexity of the SUS, distance professional qualification is an alternative to overcoming geographic barriers and providing flexible learning that accommodates professionals’ schedules ^10^. However, this teaching modality has a crucial limitation: high dropout rates, which may reach 75% ^11^. Even so, few studies have investigated the factors influencing completion of distance qualification courses, particularly those focused on obesity management ^12^^,^^13^.

This study aimed to identify the individual and contextual factors associated with the completion of a distance professional qualification course focused on the collective management of obesity in the SUS.

## Métodos

Study design, setting, and participants

This was a cross-sectional study conducted with health professionals and students residing in all States and the Federal District of Brazil who enrolled in and accessed the Professional Qualification for Obesity Management in the SUS course. This course was offered by the Research Group on Nutrition Interventions (Department of Nutrition, School of Nursing, Federal University of Minas Gerais), in collaboration with the Ministry of Health through the Telehealth Center of the Hospital das Clínicas, Federal University of Minas Gerais.

The distance professional qualification course comprised 40 hours and was based on Ministry of Health material entitled Instructive of Collective Approach for the obesity management in SUS (Instrutivo de Abordagem Coletiva para Manejo da Obesidade no SUS) ^7^. The Moodle platform was used to offer two cohorts, in 2021 and 2022, with 2,000 places each. Enrollment occurred in two phases and initially prioritized professionals from the North, Northeast, and Central-West macro-regions, followed by those working in the South and Southeast macro-regions. This difference in the course enrollment period aimed to promote equity in access to professional qualification throughout the country.

The course consisted of theoretical presentations delivered through interactive video materials and practical activities, accompanied by trained tutors from the course’s research and development team. It comprised 14 video classes (approximately eight minutes each), five short videos (up to one minute each), four discussion forums, six interim assessments, one self-assessment, and one final course assessment, with students’ participation in activities automatically recorded by the Moodle platform.

Initially, both cohorts were scheduled to last three months, but the deadline was extended by ten months (Class 1) and five months (Class 2) to increase completion rates. Tutors monitored the modules weekly and used strategies such as emails to participants who did not complete the course, social media posts, and raffles for materials.

The professional qualification course was structured into six sequential modules. Module I focused on presentation of the course and the history and purpose of the Instructive of Collective Approach for the obesity management in SUS ^7^. In Module II, the theoretical frameworks underpinning the development of the Instructive Guide were discussed, with reflections on behavior-change approaches aimed at building autonomy among both SUS users and health professionals.

The subsequent modules focused on planning and evaluation of groups and explored practical aspects of implementing effective actions for obesity management (Module III). Barriers to be overcome and possible ways to care for users were also discussed, with a focus on the Obesity Care Strategy proposed in the Instructive Guide (Module IV).

Modules V and VI addressed the practical application of the Strategy and the development of collective actions. The course concluded with a discussion of the key questions regarding the collective approach to obesity treatment in the SUS.

Data collection and measurement

The study included individual-level information, obtained through questionnaires completed by students enrolled in the professional qualification course, and contextual-level information related to the States and the Federal District where the professionals worked.

At the individual level, upon accessing the platform, students completed a questionnaire that included sociodemographic and professional data, as well as information on prior access to obesity management materials. Weekly Moodle reports monitored students’ access to course materials, enabling follow-up on participation and identification of participants who completed the course and those who did not.

Contextual variables included population size, population density (inhabitants/km^2^), monthly per capita household income, municipal human development index, Gini coefficient, prevalence of obesity among adults, rate of health workers per 1,000 inhabitants, Family Health Strategy (Estratégia Saúde da Família, ESF) coverage, higher education institutions offering health-related programs, and total annual health expenditures (BRL/inhabitant) in the respective states and the Federal District where the students worked.

The data on population size, demographic density, monthly per capita household income, and the Gini coefficient were obtained from the 2022 Population Census, made available in the Cidades@ system, linked to the Brazilian Institute of Geography and Statistics (Instituto Brasileiro de Geografia e Estatística, IBGE) ^14^. The municipal human development index was obtained from the Atlas of Human Development in Brazil website ^15^. The rate of health professionals was obtained from the 2022 National Health Workforce Census ^16^. Data on Family Health Strategy coverage for 2021 were obtained from the Institute for Health Policy Studies (Instituto de Estudos para Políticas de Saúde, IEPS) ^17^.

The prevalence of obesity was obtained from the 2023 Surveillance of Risk and Protective Factors for Chronic Diseases by Telephone Survey (Vigilância de Fatores de Risco e Proteção para Doenças Crônicas por Inquérito Telefônico, Vigitel) for the capitals of the respective States and the Federal District ^3^. The number of higher education institutions offering health-related programs was obtained from the 2023 Higher Education Census ^18^, and data on total health expenditures in 2022 were obtained from the Information System on Public Health Budgets (Sistema de Informações sobre Orçamentos Públicos em Saúde, SIOPS) ^19^.

Study variables

The study outcome variable was completion of the professional qualification course, with participants categorized as having completed or not completed the course. Participants were considered to have completed the course if they answered the initial questionnaire, completed all modules, and answered the final course evaluation questionnaire, thereby receiving a certificate.

Explanatory variables related to the individual perspective included: sex (male, female), age (years), profession (dietitian, nurse, undergraduate student, other), educational level (complete secondary education, complete higher education, specialization, residency, master’s degree, doctoral degree), employment relationship (temporary contract, formal employment contract, civil service position, other), level of care in which they worked (primary, specialized, both), length of practice in the SUS (years), weekly workload (hours), previous participation in qualification activities on the topic (no, yes), access to printed/digital materials on the topic (no, yes), and use of materials to support actions on the topic (no, yes). Undergraduate students did not answer questions about the level of care and the length of work in the SUS.

Contextual explanatory variables included: macro-region of work (North, Northeast, Midwest, Southeast, South), residence in the States and Federal District capitals (no, yes) or in metropolitan areas (no, yes), and State and Federal District-level data, such as total population (million inhabitants), population density (tertiles: 2.53-35.02; 35.03-59.05; 59.06-489.01), monthly per capita household income (in BRL), prevalence of obesity (percentage), rate of health workers per 1,000 inhabitants, Family Health Strategy coverage (percentage), higher education institutions offering health-related programs, and total annual health expenditures (tertiles: BRL 501.72-BRL 584.61; BRL 584.62-BRL 598.64; BRL 598.65-BRL 2,059.13). The municipal human development index and the Gini coefficient, both ranging from zero to one, were reported as means. Higher municipal human development index values indicated better development, whereas higher Gini coefficient values reflected greater income inequality ^14^^,^^15^.

Data analysis

The data were organized in Excel and analyzed using Stata, version 14.0, considering both the individual and contextual levels. Categorical variables were presented as relative frequencies and 95% confidence intervals (95%CI); nonparametric continuous variables as medians and interquartile ranges (IQR); and parametric continuous variables as means and 95%CI. The distribution of variables was assessed using the Shapiro-Wilk test.

Differences between participants who completed and did not complete the course were assessed using the chi-square/Fisher’s exact tests (categorical variables), Student’s t-test (parametric variables: population, per capita income, human development index, Gini coefficient, rate of health workers, and higher education institutions offering health-related programs), and the Mann-Whitney U test (non-parametric variables: age, weekly workload, and length of work in the SUS).

The association between individual and contextual factors and completion of the professional qualification course was initially assessed using multilevel logistic regression, with the States and the Federal District as the clustering units, including variables with a p-value<0.20 in the bivariate analysis. However, the model showed random-effect variance close to zero and multiple interactions, which precluded convergence. Therefore, multivariate logistic regression was used, considering only individual factors, and adjusted odds ratios (OR) and 95% confidence intervals (95%CI) were presented.

Covariates with p-value<0.20 in the bivariate analysis were included in the regression model, which was adjusted for potential confounders (age, sex, and macro-region of work). Backward elimination was used, with the following models: (i) the initial model, which included all explanatory and adjustment variables, with sequential removal of non-significant variables starting with those with the highest p-values; and (ii) the final model, which included statistically significant explanatory variables (p-value<0.05). The variables educational level, use of materials to support actions on the topic, employment relationship, length of work in the SUS, and previous participation in qualification activities on the topic were removed, in this order, to define the final model.

## Results

Of the total number of places offered for the professional qualification course (n=4,000), 3,975 students had their enrollment validated. Of these, 1,620 (40.8%) completed the initial questionnaire and were included in this study. Most participants were health professionals (84.1%) and women (91.0%) (Table 1), with a median age of 35.2 years (IQR 34.7; 35.7), with no difference between participants who completed and did not complete the course (35.4 [34.8; 36.0] versus 34.9 [34.2; 35.6]; p-value 0.306).

Regarding professional characteristics, 56.7% were dietitians, 40.9% had completed a specialization or residency program, and 30.7% were civil servants. Most worked in Primary Health Care (72.4%), 24.8% had previously participated in another professional qualification course on obesity, and 53.3% had prior access to printed/digital materials on the topic, of whom 58.3% used these materials in their professional practice (Table 1). The median weekly workload was 25.8 hours (IQR 24.9; 26.6), with no difference between participants who completed the course and those who did not (25.7 [24.6; 26.8] vs 25.9 [24.6; 27.2]; p-value 0.827). The median length of work in the SUS was 1.0 year (IQR 0.0; 8.0), and was higher among participants who completed the course (2.0 [0.0; 9.0] vs. 1.0 [0.0; 7.0]; p-value 0.036).

The course completion rate was 55.9% and was higher among dietitians (59.8% vs 52.7%; p-value 0.026), civil servants (33.5% vs. 27.0%; p-value 0.013), and participants with longer work experience in the SUS (2.0 vs. 1.0; p-value 0.036). Among participants who did not complete the course, there was a higher frequency of nurses (11.3% vs. 8.6%; p-value 0.026) and participants with other types of employment relationships (44.0% vs. 37.9%; p-value 0.013) (Table 1).

Most enrolled participants worked in the Southeast macro-region (54.8%); did not live in the capitals of the states and the Federal District (60.2%), but rather in metropolitan areas (51.0%); and resided in states and the Federal District in the first tertiles of population density (56.9%) and total annual health expenditures per inhabitant (59.7%). Among participants who completed the course, a higher proportion resided in states and the Federal District with higher population density (35.1% vs. 29.8%; p-value 0.045) and higher health expenditures (25.9% vs. 20.8%; p-value 0.018) (Table 2). The mean municipal human development index of the states and the Federal District where participants worked was 0.754 (0.752; 0.756), and the mean Gini coefficient was 0.512 (0.511; 0.514) (Table 3).

**Table 1:** Relative frequency and 95% confidence interval (95%CI) of individual level variables according to the completion of the Professional Qualification for Obesity Management in the Brazilian Unified Health System course. Brazil, 2021-2022 (n=1,620)

Variable	Total	Participants who completed the course	Participants who did not complete the course (n=714)	p-value
	(n=1,620)	(n=906)		
	% (95%CI)	% (95%CI)	% (95%CI)	
Female sex	91.0 (89.6; 92.3)	91.4 (89.4; 93.0)	90.6 (88.2; 92.5)	0.588
Profession				0.026
Dietitians	56.7 (54.2; 59.1)	59.8 (56.6; 63.0)	52.7 (49.0; 56.3)	
Nurse	9.8 (8.5; 11.4)	8.6 (6.9; 10.6)	11.3 (9.2; 13.9)	
Undergraduate student	15.9 (14.2; 17.8)	15.3 (13.1; 17.8)	16.7 (14.1; 19.6)	
Other professions	17.6 (15.8; 19.5)	16.2 (14.0; 18.8)	19.3 (16.6; 22.4)	
Educational level				0.096
Complete high school	19.7 (17.9; 21.8)	18.8 (16.3; 21.4)	21.0 (18.2; 24.2)	
Complete higher education	26.0 (23.9; 28.2)	24.3 (21.6; 27.2)	28.1 (25.0; 31.6)	
Specialization/residency	40.9 (38.5; 43.3)	43.3 (40.1; 46.5)	37.8 (34.3; 41.4)	
Master’s/doctoral degree	13.4 (11.8; 15.1)	13.7 (11.6; 16.1)	13.0 (10.7; 15.7)	
Employment relationship				0.013
Temporary contract	17.0 (15.3; 18.9)	17.7 (15.3; 20.3)	16.2 (13.7; 19.1)	
Formal employment contract	11.7 (10.2; 13.4)	10.9 (9.0; 13.1)	12.7 (10.5; 15.4)	
Civil service position	30.7 (28.5; 33.0)	33.5 (30.5; 36.7)	27.0 (23.9; 30.4)	
Other	40.6 (38.2; 43.0)	37.9 (34.7; 41.1)	44.0 (40.4; 47.6)	
Level of care in which they work^a^				0.212
Primary	72.4 (69.4; 75.2)	70.2 (66.2; 73.9)	75.4 (70.9; 79.3)	
Specialized	15.6 (13.4; 18.1)	16.8 (13.9; 20.3)	13.9 (10.9; 17.7)	
Both	12.0 (10.1; 14.2)	13.0 (10.4; 16.1)	10.7 (8.0; 14.1)	
Previous participation in professional qualification on the topic	24.8 (22.8; 27.0)	28.0 (25.2; 31.0)	20.7 (17.9; 23.9)	0.001
Access to printed/digital materials on the topic^b^	53.3 (50.9; 55.8)	56.5 (53.2; 59.7)	49.3 (45.6; 53.0)	0.004
Use of materials to support actions on the topic	58.3 (55.9; 60.7)	61.0 (57.8; 64.2)	54.9 (51.2; 58.5)	0.013

^a^ **678 missing data;** ^b^ **26 missing data.**

After adjustment in the multivariate logistic regression model, being a dietitians increased the odds of completing the professional qualification course by 55.0% (OR 1.55; 95%CI 1.09; 2.19), and having prior access to printed/digital materials on the topic increased the odds by 27.0% (OR 1.27; 95%CI 1.04; 1.56) (Table 4).

## Discusion

Completion of the distance professional qualification course for obesity management in the SUS was associated with being a dietitian and having prior access to printed/digital materials on the topic. This indicated that the field of practice and the availability of materials to support participation in the distance professional qualification course directly influence engagement, retention, and course completion.

Some limitations should be considered. The lack of individual-level data collection at enrollment led to a substantial loss of observations, underscoring the need to investigate factors associated with initial access and early engagement in qualification courses. Despite priority being given to professionals and undergraduate students from the North, Northeast, and Central-West macro-regions, participants from the South and Southeast predominated, suggesting the need for additional strategies to expand equitable access to qualification courses in the country, such as targeted dissemination, technological support, and institutional incentives.

It was not possible to perform the multilevel regression analysis as originally proposed, which limited a more detailed understanding of the simultaneous influence of individual and contextual factors on course completion. However, this may indicate that, for completion of distance qualification courses, individual factors or even micro-contextual factors, such as those related to the environment of health centers, may be more important. In this sense, further studies are recommended to identify the role of macro- and micro-contexts in professional qualification within the SUS.

**Table 2:** Relative frequencies and 95% confidence intervals (95%CI) of contextual-level variables according to completion of the Professional Qualification for Obesity Management in the Brazilian Unified Health System course. Brazil, 2021-2022 (n=1,620)

Variable	Total	Participants who completed the course (n=906)	Participants who did not complete the course (n=714)	p-value
	(n=1,620)			
	% (95%CI)	% (95%CI)	% (95%CI)	
Macro-region of work				0.097
North	6.6 (5.5; 7.9)	6.2 (4.8; 7.9)	7.1 (5.5; 9.3)	
Northeast	23.5 (21.5; 25.6)	24.3 (21.6; 27.2)	22.5 (19.6; 25.8)	
Central-West	3.6 (2.8; 4.6)	2.6 (1.8; 3.9)	4.8 (3.4; 6.6)	
Southeast	54.8 (52.4; 57.2)	54.4 (51.1; 57.6)	55.3 (51.6; 58.9)	
South	11.5 (10.0; 13.1)	12.5 (10.5; 14.8)	10.2 (8.2; 12.7)	
Lives in the capitals of the states and the Federal District^a^	39.8 (37.4; 42.3)	38.2 (35.0; 41.5)	41.9 (38.2; 45.7)	0.137
Lives in a metropolitan area^a^	51.0 (48.5; 53.5)	49.8 (46.4; 53.1)	52.5 (48.7; 56.2)	0.287
Population density (inhabitants / km^2^)				0.045
2,53-35,02	56.9 (54.5; 59.4)	54.3 (51.0; 57.5)	60.4 (56.7; 63.9)	
35,03-59,05	10.2 (8.9; 11.8)	10.6 (8.7; 12.8)	9.8 (7.8; 12.2)	
59,06-489,01	32.8 (30.5; 35.1)	35.1 (32.0; 38.3)	29.8 (26.6; 33.3)	
Prevalence of obesity in 2023	23.2 (23.1; 23.3)	23.1 (23.0; 23.3)	23.3 (23.1; 23.5)	0.423
Family Health Strategy coverage	70.4 (69.7; 71.2)	70.3 (69.3; 71.4)	70.5 (69.4; 71.7)	0.810
Total annual health expenditure (BRL/inhabitant)				0.018
501,72-584,61	59.7 (57.3; 62.1)	56.8 (53.5; 60.0)	63.5 (59.9; 67.0)	
584,62-598,64	16.6 (14.9; 18.5)	17.3 (15.0; 20.0)	15.7 (13.2; 18.5)	
598,65-2059,13	23.7 (21.6; 25.8)	25.9 (23.1; 28.9)	20.8 (17.9; 24.0)	

a **86 missing data.**

Although contextual factors could not be analyzed jointly with individual factors, the findings may still provide useful information for health managers. A higher proportion of participants who completed the course worked in municipalities with higher population density and higher health expenditures. These differences may be explained by the fact that most participants who completed the course worked in the Southeast macro-region and in metropolitan areas. A similar pattern was observed in self-instructional courses offered by the Open University of the Brazilian Unified Health System (Universidade Aberta do Sistema Único de Saúde, UNA-SUS) ^20^ and in professional qualification focused on collective actions to promote healthy, adequate eating ^11^.

States and the Federal District with higher population densities and better infrastructure tend to offer better internet access conditions, which may positively influence participation in and completion of distance learning courses ^21^. In Brazil, there are substantial disparities in internet availability, with greater communication infrastructure density in the Southeast and South macro-regions and lower density in the North and Northeast macro-regions ^22^. This scenario reinforces the need for public policies to ensure equitable access to distance education qualification courses nationwide, including greater digital inclusion across all macro-regions.

**Table 3:** Means and 95% confidence intervals (95%CI) of contextual-level variables according to completion of the Professional Qualification for Obesity Management in the Brazilian Unified Health System course. Brazil, 2021-2022 (n=1,620)

Variable	Total	Participants who completed the course (n=906)	Participants who did not complete the course (n=714)	p-value
	(n=1,620)			
	Mean (95%CI)	Mean (95%CI)	Mean (95%CI)	
Total population (million inhabitants)	17,1	17	17,2	0.748
	(16,500; 17,700)	(16,200; 17,800)	(16,400; 18,000)	
Monthly per capita household income (BRL)	1,559.81	1,558.54	1,561.43	0.890
	(1,539.43; 1,580.20)	(1,531.49; 1,585.59)	(1,530.35; 1,592.50)	
Municipal Human Development Index	0.754 (0.752; 0.756)	0.755 (0.752; 0.757)	0.753 (0.751; 0.756)	0.596
Gini coefficient	0.512 (0.511; 0.514)	0.512 (0.510; 0.515)	0.513 (0.510; 0.515)	0.790
Health workers rate (per 1,000 inhabitants)	1.27 (1.23; 1.32)	1.28 (1.22; 1.34)	1.27 (1.20; 1.33)	0.807
Higher education institution offering health-related courses	180.2 (177.3; 183.1)	180.6 (177.0; 184.2)	179.3 (174.3; 184.3)	0.679

**Table 4:** Odds ratios (OR) and 95% confidence intervals (95%CI) from multivariate logistic regression for completion of the Professional Qualification for Obesity Management in the Brazilian Unified Health System course. Brazil, 2021-2022 (n=1,620)

Variable	Initial model	Final model
	OR (95%CI)	OR (95%CI)
Profession		
Nurse	1.00	1.00
Dietitian	1.63 (1.13; 2.34)	1.55 (1.09; 2.19)
Undergraduate student	1.18 (0.78; 1.77)	1.09 (0.74; 1.64)
Other	1.46 (0.81; 2.64)	1.36 (0.89; 2.10)
Educational level		
Complete high school	1.00	-
Complete higher education	0.83 (0.57; 1.21)	-
Specialization/residency	1.09 (0.78; 1.52)	-
Postgraduate degree	0.83 (0.61; 1.14)	-
Employment relationship		
Temporary contract	1.00	-
Formal employment contract	0.83 (0.56; 1.21)	-
Civil service position	1.09 (0.78; 1.52)	-
Other	0.83 (0.61; 1.14)	-
Length of practice in the Brazilian Unified Health System (Sistema Único de Saúde, SUS)	1.01 (0.99; 1.03)	-
Previous participation in professional qualification activities on the topic	1.18 (0.91; 1.54)	-
Access to printed/digital materials on the topic	1.16 (0.90; 1.49)	1.27 (1.04; 1.56)
Use of materials to support actions on the topic	1.0 (0.77; 1.29)	-

Among the factors associated with course completion, being a dietitian was important. The close relationship between obesity and nutrition may have contributed to these findings. However, due to the multifactorial characteristic of obesity, this is a multiprofessional and intersectoral issue ^7^^,^^23^that requires the involvement of different health professional categories, especially physicians, nurses, dietitian, psychologists, and physical education professionals. To make this possible, institutional support and adequate infrastructure are essential, including protected working hours for continuing education, high-quality access to technological resources, and recognition of qualification for career progression.

Different professional categories need qualification to address the challenges of obesity management, especially when conducting therapeutic groups. A web-based quantitative exploratory study conducted in 2018 with 1,323 Brazilian health professionals showed that 72% of respondents agreed with the statement, “It is very difficult to treat individuals with obesity.” More than half of the respondents reported that the lack of qualification negatively affected the effective implementation of actions (75%) and that the time available for planning collective actions was insufficient (61%). For nurses, group care for individuals with obesity was the most difficult approach to implement (89%), compared with 49% among dietitian ^8^.

In addition to the professional category, participants who had prior access to printed/digital materials on obesity management were more likely to complete the course. This finding reinforces the importance of making educational resources available and continuously promoting continuing education in health services to foster engagement and sustain learning ^24^. The progressive increase in obesity prevalence among Brazilian adults ^4^, and consequently the failure to achieve the goal of halting its growth by 2022 ^25^, reinforces the urgency of expanding the visibility and availability of instructional materials, particularly printed materials, given the limitations in digital access in health centers. The broad provision of professional qualification courses across the entire Health Care Network is necessary to support an adequate response to this public health challenge.

The scarcity of instructional materials available to health professionals may be related to weaknesses in the articulation among continuing education, surveillance, and health care, which should work together to produce and disseminate these resources. In Brazil, despite the growing political emphasis on obesity ^7^^,^^20^^,^^26^^,^^27^^,^^28^, the availability of effective support materials in health services and access to qualification courses remain limited. Among these challenges are the scarcity of resources for printing and distributing printed materials, the insufficient visibility and dissemination of digital materials, and their limited use in health centers due to connectivity constraints, which also hinder participation in distance learning courses ^8^.

Among the strengths of this study was its focus on factors associated with completion of a distance professional qualification course for obesity management in the SUS, encompassing all macro-regions and all states and the Federal District. Its findings have the potential to support the improvement of future professional qualification initiatives offered by the Ministry of Health, as well as to inform government strategies aimed at strengthening continuing education in health, with a view to improving the qualification of SUS health professionals and expanding the capacity of the Health Care Network to address the obesity epidemic in Brazil.

Although participation in professional qualification courses is central to the continuous improvement of longitudinal care for SUS users, persistent challenges remain, including insufficient managerial support and the resulting professional demotivation, as well as work overload, which hinder participation in and completion of courses ^11^^,^^29^. This was evident in the present study, which showed that approximately 60% of enrolled participants did not access the Moodle platform to begin the course.

To change this scenario, managerial support is essential to foster engagement by ensuring protected study time, adequate infrastructure, and formal recognition of participation in qualification courses. Employment stability may facilitate the pursuit of professional development. Civil servants covered by SUS Career, Position, and Salary Plans that include continuing education as a criterion for career progression ^30^ may have a greater interest in and capacity to complete qualification courses. This was reflected in the present study, in which most participants who completed the course were civil servants and had longer experience working in the SUS. In addition, strategies are needed to promote equity among the states and the Federal District and across the country’s macro-regions.

The findings of this study showed that being a dietitian and having prior access to printed/digital materials increased the odds of completing the professional qualification activity for obesity management. These findings highlight the need to encourage participation from other professional categories in qualification activities on this topic, including the prior dissemination of materials, to maximize the impact of continuing education on professional practice and the quality of care provided to individuals with obesity in the SUS.

## Data Availability

The contents of the database are available upon request to the corresponding author.
