# Supplementary material for: Observational study of the coverage rate and survival analysis of COVID-19 vaccination among Indigenous people in Roraima, 2021-2023 Individual and contextual factors and professional qualification completion for obesity management: a cross-sectional study, Brazil, 2021-2022
Source: Epidemiol Serv Saude. 2026 Jul 16;35:e20251154. doi: 10.1590/S2237-96222026v35e20251154.en (PMC13686389; doi:10.1590/S2237-96222026v35e20251154.en)
Supplement: Translation

# Translation: Fatores individuais e contextuais, e conclusão de qualificação profissional para o manejo da obesidade: estudo transversal, Brasil, 2021-2022

**Authors:** Angélica Ribeiro e Silva, Nathália Luíza Ferreira, Patrícia Pinheiro de Freitas, Mariana Souza Lopes, Aline Cristine Souza Lopes

**Affiliations:** 1 Universidade Federal de Minas Gerais, Departamento de Nutrição, Belo Horizonte, MG, Brasil Universidade Federal de Minas Gerais Departamento de Nutrição Belo Horizonte MG Brasil; 2 Universidade Federal de Lavras, Departamento de Nutrição, Lavras, MG, Brasil Universidade Federal de Lavras Departamento de Nutrição Lavras MG Brasil; 3 Universidade Federal dos Vales do Jequitinhonha e Mucuri, Departamento de Nutrição, Diamantina, MG, Brasil Universidade Federal dos Vales do Jequitinhonha e Mucuri Departamento de Nutrição Diamantina MG Brasil; 4 Universidade Federal da Paraíba, Departamento de Nutrição, João Pessoa, PB, Brasil Universidade Federal da Paraíba Departamento de Nutrição João Pessoa PB Brasil

**Keywords:** Obesidade, Educação Continuada, Educação Profissional em Saúde Pública, Sistema Único de Saúde, Estudos Transversais

## Abstract

**Objetivo:**

Identificar os fatores individuais e contextuais associados à conclusão de qualificação profissional a distância para o manejo coletivo da obesidade no Sistema Único de Saúde.

**Métodos:**

Estudo transversal com profissionais e estudantes da área da saúde. O curso, com carga horária de 40 horas, contou com tutoria e foi baseado em material do Ministério da Saúde. Os fatores individuais analisados incluíram dados sociodemográficos e profissionais obtidos no início do curso; e os contextuais abarcaram dados econômicos e demográficos das Unidades da Federação de atuação, obtidos em sites institucionais. Participantes que realizaram todas as atividades foram considerados concluintes. Devido à falha de convergência das variáveis contextuais na regressão logística multinível, realizou-se regressão logística multivariada, incluindo razão de chances (odds ratio, OR) ajustada e intervalo de confiança de 95% (IC95%) por sexo, idade e macrorregião do país.

**Resultados:**

Foram 3.975 inscritos no curso; 1.620 (40,8%) acessaram a plataforma virtual. A maioria era de mulheres (91,0%) e nutricionistas (56,7%) e atuava na macrorregião Sudeste do país (54,8%) e regiões metropolitanas (51,0%). Após ajustes do modelo, verificou-se que ser nutricionista (OR 1,55; IC95% 1,09; 2,19) e ter acesso prévio a materiais impressos/digitais sobre o tema (OR 1,27; IC95% 1,04; 1,56) aumentavam as chances de conclusão da qualificação profissional.

**Conclusão:**

A conclusão da qualificação profissional foi associada a ser nutricionista e ao acesso prévio a materiais impressos/digitais sobre o tema, o que evidenciou a importância da categoria profissional e de sucessivas aproximações com o tema para favorecer o engajamento e a conclusão do curso.

**Aspectos éticos**

Esta pesquisa respeitou os princípios éticos, obtendo os seguintes dados de aprovação:

Comitê de ética em pesquisa: Universidade Federal de Minas Gerais

Número do parecer: 4294291

Data de aprovação: 23/9/2020

Certificado de apresentação de apreciação ética: 93992418.0.0000.5149

Registro do consentimento livre e esclarecido: Obtido.

## Introdução

A obesidade é um dos três componentes centrais na sindemia global ^1^. Em 2022, estimou-se que mais de 800 milhões de pessoas no mundo possuíam obesidade ^2^. No Brasil, sua prevalência aumentou 13% em 17 anos ^3^, e, caso sejam mantidas as tendências atuais, poderá afetar 83 milhões de adultos em 2044 ^4^. Esse panorama gera preocupação tanto pelos potenciais impactos na saúde conferidos pela obesidade em si, quanto pelas comorbidades associadas ^5^.

A obesidade possui etiologia multifatorial, o que torna seu manejo complexo e desafiador ^1^. Embora a abordagem individual seja amplamente utilizada, a abordagem coletiva constitui importante estratégia para reduzir o peso corporal e alcançar outros benefícios, sobretudo quando integrada à abordagem individual ^6^. Intervenções em grupo favorecem o suporte social e o compartilhamento de vivências, o que promove maior continuidade do tratamento e potencialmente maior efetividade na manutenção da redução de peso em longo prazo, quando comparadas à abordagem individual ^6^. A abordagem coletiva constitui um ganho importante no contexto da saúde pública, por alcançar simultaneamente um número maior de indivíduos ^7^.

Conteúdos sobre abordagem coletiva para o manejo da obesidade são ainda escassos nos cursos de qualificação profissional da área da saúde e nos currículos dos cursos da área da saúde no Brasil. Como consequência, profissionais de saúde que atuam no Sistema Único de Saúde (SUS) apontam como dificuldades, para o desenvolvimento de grupos, a ausência ou o acesso insuficiente a cursos de qualificação profissional e materiais instrucionais ^8^. Para enfrentar esse cenário, é essencial que os profissionais sejam continuamente qualificados por cursos baseados em evidências científicas atuais e alinhados às diretrizes e às necessidades do SUS, conforme preconizado pela Política Nacional de Educação Permanente em Saúde ^9^.

Considerando a extensão territorial do Brasil e a complexidade do SUS, a qualificação profissional a distância é uma alternativa para superar as barreiras geográficas e proporcionar o aprendizado flexível à agenda dos profissionais ^10^. Entretanto, essa modalidade de ensino apresenta uma limitação crucial: as elevadas taxas de abandono, que podem chegar a 75% ^11^. Ainda assim, poucos estudos investigam os fatores que influenciam a conclusão de cursos de qualificação a distância, sobretudo voltados para o manejo da obesidade ^12^^,^^13^.

Este estudo objetivou identificar os fatores individuais e contextuais relacionados à conclusão de curso de qualificação profissional a distância voltado para o manejo coletivo da obesidade no SUS.

## Métodos

Delineamento, contexto e participantes do estudo

Trata-se de estudo transversal realizado com profissionais e estudantes da área de saúde residentes em todas as Unidades da Federação (UF) do Brasil, que se inscreveram e acessaram o curso Qualificação Profissional para Manejo da Obesidade no SUS. Esse curso foi oferecido pelo Grupo de Pesquisa de Intervenções em Nutrição (Departamento de Nutrição, Escola de Enfermagem da Universidade Federal de Minas Gerais), em colaboração com o Ministério da Saúde por meio do Telessaúde do Hospital das Clínicas da Universidade Federal de Minas Gerais.

O curso de qualificação profissional a distância contou com carga horária de 40 horas e foi baseado em material do Ministério da Saúde denominado Instrutivo de Abordagem Coletiva para Manejo da Obesidade no SUS ^7^. Utilizou-se a plataforma Moodle para ofertar, em 2021 e 2022, duas turmas com 2 mil vagas cada. As inscrições ocorreram em duas chamadas e priorizaram inicialmente profissionais das macrorregiões Norte, Nordeste e Centro-Oeste, e, em seguida, aqueles que atuavam nas macrorregiões Sul e Sudeste. Essa diferenciação no período de inscrição no curso objetivou promover a equidade no acesso às qualificações profissionais no país.

O curso constou de exposições teóricas veiculadas por vídeos interativos do tipo videografismo e de atividades práticas acompanhadas por tutores treinados que integravam a equipe de pesquisa e de desenvolvimento do curso. Foi composto por 14 videoaulas (cerca de oito minutos cada), cinco vídeos curtos (até um minuto cada), quatro fóruns de discussão, seis avaliações parciais, uma autoavaliação e uma avaliação final do curso, sendo a participação dos alunos nas atividades registrada automaticamente pela plataforma Moodle.

Inicialmente, foram previstos três meses de duração para ambas as turmas, mas o prazo foi estendido em dez meses (turma 1) e cinco meses (turma 2) para ampliar as conclusões. As tutoras monitoravam semanalmente os módulos e usavam estratégias como e-mails aos não concluintes, postagens em redes sociais e sorteio de materiais.

O curso de qualificação profissional foi estruturado em seis módulos sequenciais. O módulo I focou a apresentação do curso e o histórico e propósito do Instrutivo de Abordagem Coletiva para Manejo da Obesidade no SUS ^7^. No módulo II, foram discutidos os referenciais teóricos que embasaram a elaboração do Instrutivo, com reflexões sobre abordagens para a mudança de comportamento voltadas para construir a autonomia tanto dos usuários do SUS quanto dos profissionais de saúde.

Os módulos subsequentes concentraram-se no planejamento e na avaliação dos grupos e exploraram os aspectos práticos para implementar ações efetivas no manejo da obesidade (módulo III). Também foram discutidos as barreiras a serem superadas e os possíveis caminhos para cuidar do usuário, com foco na Estratégia de Cuidado da Pessoa com Obesidade proposta pelo Instrutivo (módulo IV).

Os módulos V e VI abordaram a aplicação prática da Estratégia e o desenvolvimento de ações coletivas. O curso foi concluído com a discussão das principais dúvidas relacionadas à abordagem coletiva para o tratamento da obesidade no SUS.

Coleta e mensuração dos dados

A pesquisa contemplou informações do âmbito individual, obtidas por questionários respondidos pelos alunos inscritos no curso de qualificação profissional, e informações de nível contextual, referentes às UF de atuação dos profissionais.

No âmbito individual, ao ingressar na plataforma, os alunos preencheram questionário contendo dados sociodemográficos e profissionais, com relação ao acesso prévio a materiais sobre manejo da obesidade. Relatórios semanais do Moodle monitoravam o acesso dos alunos aos materiais, o que permitiu acompanhar a participação e identificar concluintes e não concluintes.

As variáveis do contexto incluíram o tamanho da população, a densidade demográfica (habitantes/km2), a renda mensal domiciliar per capita, o índice de desenvolvimento humano municipal, o coeficiente de Gini, a prevalência de obesidade entre adultos, a taxa de trabalhadores de saúde por mil habitantes, a cobertura da Estratégia Saúde da Família, as instituições de ensino superior com cursos da área da saúde e as despesas anuais totais com saúde (R$/habitante) das respectivas UF de atuação dos alunos.

Os dados relativos ao tamanho da população, à densidade demográfica, à renda domiciliar mensal per capita e ao coeficiente de Gini foram obtidos no Censo Demográfico 2022, disponibilizados no sistema Cidades@, vinculado ao Instituto Brasileiro de Geografia e Estatística ^14^. O índice de desenvolvimento humano municipal foi extraído no site do Atlas do Desenvolvimento Humano no Brasil ^15^. A taxa de profissionais de saúde foi obtida a partir do Censo Nacional da Força de Trabalho na Saúde de 2022 ^16^. Para a análise da cobertura da Estratégia de Saúde da Família, consultaram-se dados do Instituto de Estudos para Políticas de Saúde relativos a 2021 ^17^.

A prevalência de obesidade foi obtida a partir do Sistema de Vigilância de Fatores de Risco e Proteção para Doenças Crônicas por Inquérito Telefônico para as capitais das respectivas UF em 2023 ^3^. O número de instituições de ensino superior com cursos da área da saúde foi obtido a partir do Censo da Educação Superior 2023 ^18^, e a procedência dos dados sobre o total de despesas em saúde em 2022, a partir do Sistema de Informações sobre Orçamentos Públicos em Saúde ^19^.

Variáveis do estudo

A variável desfecho do estudo foi a conclusão da qualificação profissional, sendo os participantes categorizados como concluintes e não concluintes. Foi considerado como concluinte o aluno que respondeu ao questionário inicial, concluiu todos os módulos e respondeu ao questionário de avaliação final do curso, obtendo a certificação.

As variáveis explicativas relacionadas à perspectiva individual incluíram: sexo (masculino, feminino), idade (anos), profissão (nutricionista, enfermeiro, graduando, outras), escolaridade (ensino médio completo, ensino superior completo, especialização, residência, mestrado, doutorado), vínculo empregatício (contrato temporário, carteira de trabalho assinada, concurso público, outro), nível de atenção em que atua (primária, especializada, ambas), tempo de atuação no SUS (anos), jornada de trabalho semanal (horas), participação prévia em atividades de qualificação sobre o tema (não, sim), acesso a materiais impressos/digitais sobre o tema (não, sim) e uso de materiais para apoiar ações sobre o tema (não, sim). Os graduandos não respondiam às questões relacionadas ao nível e ao tempo de atuação no SUS.

**Tabela 1:** Frequência relativa e intervalo de confiança de 95% (IC95%) das variáveis de nível individual segundo a conclusão no curso de Qualificação para Manejo da Obesidade no Sistema Único de Saúde. Brasil, 2021-2022 (n=1.620)

Variável	Total	Concluintes	Não concluintes (n=714)	p-valor
	(n=1,620)	(n=906)		
	% (IC95%)	% (IC95%)	% (IC95%)	
Sexo feminino	91,0 (89,6; 92,3)	91,4 (89,4; 93,0)	90,6 (88,2; 92,5)	0,588
Profissão				0,026
Nutricionista	56,7 (54,2; 59,1)	59,8 (56,6; 63,0)	52,7 (49,0; 56,3)	
Enfermeiro	9,8 (8,5; 11,4)	8,6 (6,9; 10,6)	11,3 (9,2; 13,9)	
Graduando	15,9 (14,2; 17,8)	15,3 (13,1; 17,8)	16,7 (14,1; 19,6)	
Outras profissões	17,6 (15,8; 19,5)	16,2 (14,0; 18,8)	19,3 (16,6; 22,4)	
Escolaridade				0,096
Ensino médio completo	19,7 (17,9; 21,8)	18,8 (16,3; 21,4)	21,0 (18,2; 24,2)	
Ensino superior completo	26,0 (23,9; 28,2)	24,3 (21,6; 27,2)	28,1 (25,0; 31,6)	
Especialização/residência	40,9 (38,5; 43,3)	43,3 (40,1; 46,5)	37,8 (34,3; 41,4)	
Mestrado/doutorado	13,4 (11,8; 15,1)	13,7 (11,6; 16,1)	13,0 (10,7; 15,7)	
Vínculo empregatício				0,013
Contrato temporário	17,0 (15,3; 18,9)	17,7 (15,3; 20,3)	16,2 (13,7; 19,1)	
Carteira de trabalho assinada	11,7 (10,2; 13,4)	10,9 (9,0; 13,1)	12,7 (10,5; 15,4)	
Concurso público	30,7 (28,5; 33,0)	33,5 (30,5; 36,7)	27,0 (23,9; 30,4)	
Outro	40,6 (38,2; 43,0)	37,9 (34,7; 41,1)	44,0 (40,4; 47,6)	
Nível de atenção em que atuaa				0,212
Primária	72,4 (69,4; 75,2)	70,2 (66,2; 73,9)	75,4 (70,9; 79,3)	
Especializada	15,6 (13,4; 18,1)	16,8 (13,9; 20,3)	13,9 (10,9; 17,7)	
Ambas	12,0 (10,1; 14,2)	13,0 (10,4; 16,1)	10,7 (8,0; 14,1)	
Participação prévia em qualificação profissional sobre o tema	24,8 (22,8; 27,0)	28,0 (25,2; 31,0)	20,7 (17,9; 23,9)	0,001
Acesso a materiais impressos/digitais sobre o temab	53,3 (50,9; 55,8)	56,5 (53,2; 59,7)	49,3 (45,6; 53,0)	0,004
Uso de materiais para apoiar ações sobre o tema	58,3 (55,9; 60,7)	61,0 (57,8; 64,2)	54,9 (51,2; 58,5)	0,013

a 678 dados faltantes; ^b^26 dados faltantes.

As variáveis explicativas contextuais constaram de: macrorregião de atuação (Norte, Nordeste, Centro-Oeste, Sudeste, Sul), residir nas capitais das UF (não, sim) ou em região metropolitana (não, sim), dados relativos à UF de atuação, como população total (milhão de habitantes), densidade demográfica (tercis: 2,53-35,02; 35,03-59,05; 59,06-489,01), renda mensal domiciliar per capita (reais), prevalência de obesidade (porcentagem), taxa de trabalhadores de saúde a cada mil habitantes, cobertura da Estratégia de Saúde da Família (porcentagem), instituições de ensino superior com curso na área da saúde e total de despesas anuais em saúde (tercis: R$ 501,72-R$ 584,61; R$ 584,62-R$ 598,64; R$ 598,65-R$ 2.059,13). O índice de desenvolvimento humano municipal e o coeficiente de Gini, que variam de zero a um, foram expressos como médias. Maiores valores de índice de desenvolvimento humano municipal indicaram melhor desenvolvimento, enquanto maiores valores do coeficiente de Gini refletiram maior desigualdade de renda ^14^^,^^15^.

Análise de dados

Os dados foram organizados no programa Excel e analisados no Stata, versão 14.0, considerando os níveis individual e contextual. As variáveis categóricas foram apresentadas pela frequência relativa e pelo intervalo de confiança de 95% (IC95%); as contínuas não paramétricas pela mediana e pelo intervalo interquartílico (IIQ); e as paramétricas pela média e pelo IC95%. A distribuição das variáveis foi testada pelo teste Shapiro-Wilk.

**Tabela 2:** Frequência relativa e intervalo de confiança de 95% (IC95%) das variáveis de nível contextual segundo a conclusão no curso de Qualificação para Manejo da Obesidade no Sistema Único de Saúde. Brasil, 2021-2022 (n=1.620)

Variável	Total	Concluintes	Não concluintes	p-valor
	(n=1,620)	(n=906)	(n=714)	
	% (IC95%)	% (IC95%)	% (IC95%)	
Macrorregião de atuação				0,097
Norte	6,6 (5,5; 7,9)	6,2 (4,8; 7,9)	7,1 (5,5; 9,3)	
Nordeste	23,5 (21,5; 25,6)	24,3 (21,6; 27,2)	22,5 (19,6; 25,8)	
Centro-Oeste	3,6 (2,8; 4,6)	2,6 (1,8; 3,9)	4,8 (3,4; 6,6)	
Sudeste	54,8 (52,4; 57,2)	54,4 (51,1; 57,6)	55,3 (51,6; 58,9)	
Sul	11,5 (10,0; 13,1)	12,5 (10,5; 14,8)	10,2 (8,2; 12,7)	
Vive nas capitais das Unidades de Federação^a^	39,8 (37,4; 42,3)	38,2 (35,0; 41,5)	41,9 (38,2; 45,7)	0,137
Vivo em região Metropolitana^a^	51,0 (48,5; 53,5)	49,8 (46,4; 53,1)	52,5 (48,7; 56,2)	0,287
Densidade demográfica (habitantes/km^2^)				0,045
2,53-35,02	56,9 (54,5; 59,4)	54,3 (51,0; 57,5)	60,4 (56,7; 63,9)	
35,03-59,05	10,2 (8,9; 11,8)	10,6 (8,7; 12,8)	9,8 (7,8; 12,2)	
59,06-489,01	32,8 (30,5; 35,1)	35,1 (32,0; 38,3)	29,8 (26,6; 33,3)	
Prevalência de obesidade em 2023	23,2 (23,1; 23,3)	23,1 (23,0; 23,3)	23,3 (23,1; 23,5)	0,423
Cobertura da Estratégia de Saúde da Família	70,4 (69,7; 71,2)	70,3 (69,3; 71,4)	70,5 (69,4; 71,7)	0,81
Totais de despesas anuais com saúde (R$/habitante)				0,018
501,72-584,61	59,7 (57,3; 62,1)	56,8 (53,5; 60,0)	63,5 (59,9; 67,0)	
584,62-598,64	16,6 (14,9; 18,5)	17,3 (15,0; 20,0)	15,7 (13,2; 18,5)	
598,65-2059,13	23,7 (21,6; 25,8)	25,9 (23,1; 28,9)	20,8 (17,9; 24,0)	

a 86 dados faltantes.

As diferenças entre os alunos concluintes e não concluintes foram avaliadas pelos testes qui-quadrado/exato de Fisher (categóricas), t de Student (paramétricas: população, renda per capita, índice de desenvolvimento humano, coeficiente de Gini, taxa de trabalhadores de saúde e instituições de ensino superior com cursos de saúde) e U de Mann-Whitney (não paramétricas: idade, jornada de trabalho e tempo no SUS).

A associação entre os fatores individuais e contextuais e a conclusão da qualificação foi inicialmente testada pela regressão logística multinível, com a UF como aglomerado, incluindo variáveis com p-valor<0,20 na bivariada. Contudo, o modelo apresentou variância do efeito aleatório próxima de zero e múltiplas interações, o que inviabilizou a convergência. Assim, optou-se por empregar a regressão logística multivariada considerando apenas os fatores individuais na análise, com apresentação das razões de chances (odds ratio, OR) e IC95%.

As covariáveis com p-valor<0,20 na análise bivariada foram incluídas no modelo de regressão, ajustado por potenciais confundidores (idade, sexo e macrorregião de atuação). Utilizaram-se a estratégia de eliminação retrógrada e os seguintes modelos: (i) inicial, que incluiu todas as variáveis explicativas e de ajuste, com remoção sequencial das variáveis não significativas a partir dos maiores p-valores; e (ii) final, que incluiu as variáveis explicativas significativas (p-valor<0,05). As variáveis escolaridade, uso de materiais para apoiar ações sobre o tema, vínculo empregatício, tempo de atuação no SUS e participação prévia em atividades de qualificação sobre o tema foram retiradas, nessa ordem, para definir o modelo final.

## Resultados

Do total de vagas disponibilizadas para a qualificação profissional (n=4.000), 3.975 alunos tiveram sua inscrição validada. Destes, 1.620 (40,8%) iniciaram o curso, respondendo ao questionário inicial, sendo aqui investigados. A maioria dos alunos era profissional de saúde (84,1%), mulheres (91,0%) (Tabela 1), com mediana de idade de 35,2 anos (IIQ 34,7; 35,7), sem diferença entre os concluintes e não concluintes (35,4 [34,8; 36,0] versus 34,9 [34,2; 35,6]; p-valor 0,306).

**3:** Média e intervalo de confiança de 95% (IC95%) das variáveis de nível contextual segundo a conclusão no curso de Qualificação para Manejo da Obesidade no Sistema Único de Saúde. Brasil, 2021-2022 (n=1.620)

Variável	Total	Concluintes	Não concluintes	p-valor
	(n=1,620)	(n=906)	(n=714)	
	Média (IC95%)	Média (IC95%)	Média (IC95%)	
População total (milhão de habitantes)	17.100	17.000	17.200	0,748
	(16.500; 17.700)	(16.200; 17.800)	(16.400; 18.000)	
Renda mensal domiciliar per capita (R$)	1.559,81	1.558,54	1.561,43	0,89
	(1.539,43; 1.580,20)	(1.531,49; 1.585,59)	(1.530,35; 1.592,50)	
Índice de desenvolvimento humano municipal	0,754 (0,752; 0,756)	0,755 (0,752; 0,757)	0,753 (0,751; 0,756)	0,596
Coeficiente de Gini	0,512 (0,511; 0,514)	0,512 (0,510; 0,515)	0,513 (0,510; 0,515)	0,79
Taxa de trabalhadores da saúde (por mil habitantes)	1,27 (1,23; 1,32)	1,28 (1,22; 1,34)	1,27 (1,20; 1,33)	0,807
Instituição de ensino superior com curso de saúde	180,2 (177,3; 183,1)	180,6 (177,0; 184,2)	179,3 (174,3; 184,3)	0,679

**Tabela 4:** Razão de chances (odds ratio, OR) e intervalo de confiança de 95% (IC95%) a partir de regressão logística multivariada para conclusão do curso de Qualificação para Manejo da Obesidade no Sistema Único de Saúde. Brasil, 2021-2022 (n=1.620)

Variável	Modelo inicial OR (IC95%)	Modelo final OR (IC95%)
Profissão		
Enfermeiro	1,00	1,00
Nutricionista	1,63 (1,13; 2,34)	1,55 (1,09; 2,19)
Graduando	1,18 (0,78; 1,77)	1,09 (0,74; 1,64)
Outra	1,46 (0,81; 2,64)	1,36 (0,89; 2,10)
Escolaridade		
Médio completo	1,00	-
Superior completo	0,83 (0,57; 1,21)	-
Especialização/residência	1,09 (0,78; 1,52)	-
Pós-graduação	0,83 (0,61; 1,14)	-
Vínculo empregatício		
Contrato temporário	1,00	-
Carteira de trabalho assinado	0,83 (0,56; 1,21)	-
Concurso público	1,09 (0,78; 1,52)	-
Outro	0,83 (0,61; 1,14)	-
Tempo de atuação no Sistema Único de Saúde	1,01 (0,99; 1,03)	-
Participação prévia em atividade de qualificação profissional sobre o tema	1,18 (0,91; 1,54)	-
Acesso a materiais impressos/digitais sobre o tema	1,16 (0,90; 1,49)	1,27 (1,04; 1,56)
Uso de materiais para apoiar ações sobre o tema	1,0 (0,77; 1,29)	-

Quanto aos dados profissionais, 56,7% eram nutricionistas, 40,9% possuíam especialização/residência, e 30,7% eram servidores públicos concursados. A maioria atuava na Atenção Primária (72,4%), 24,8% dos alunos já haviam realizado outra qualificação profissional sobre obesidade, e 53,3% tinham acesso prévio a materiais impressos/digitais sobre o tema e 58,3% os utilizava na prática profissional (Tabela 1). A mediana da jornada semanal de trabalho foi 25,8 horas (IIQ 24,9; 26,6), sem diferenças entre os alunos concluintes e não concluintes (25,7 [24,6; 26,8] vs. 25,9 [24,6; 27,2]; p-valor 0,827). A mediana do tempo de atuação no SUS foi 1,0 ano (IIQ 0,0; 8,0), sendo maior entre os alunos concluintes (2,0 [0,0; 9,0] vs. 1,0 [0,0; 7,0]; p-valor 0,036).

A taxa de conclusão no curso foi 55,9%, sendo mais frequente entre os nutricionistas (59,8% vs. 52,7%; p-valor 0,026), servidores concursados (33,5% vs. 27,0%; p-valor 0,013) e com maior tempo de atuação no SUS (2,0 vs. 1,0; p-valor 0,036). Entre os alunos não concluintes, observou-se maior frequência de enfermeiros (11,3% vs. 8,6%; p-valor 0,026) e com outro vínculo empregatício (44,0% vs. 37,9%; p-valor 0,013) (Tabela 1).

A maioria dos alunos inscritos atuava na macrorregião Sudeste (54,8%); não vivia nas capitais das UF (60,2%), mas sim em regiões metropolitanas (51,0%); e residia em UF nos primeiros tercis de densidade populacional (56,9%) e de gastos totais anuais com saúde por habitante (59,7%). Entre os alunos concluintes, identificou-se maior frequência de residentes em UF com maior densidade populacional (35,1% vs. 29,8%; p-valor 0,045) e com gastos com saúde mais altos (25,9% vs. 20,8%; p-valor 0,018) (Tabela 2). A média do índice de desenvolvimento humano municipal das UF na qual os alunos atuavam foi 0,754 (0,752; 0,756) e do coeficiente de Gini 0,512 (0,511; 0,514) (Tabela 3).

Após ajustes do modelo de regressão logística multivariada, verificou-se que ser nutricionista aumentava em 55,0% as chances de concluir a qualificação profissional (OR 1,55; IC95% 1,09; 2,19) e ter acesso prévio a materiais impressos/digitais sobre o tema em 27,0% (OR 1,27; IC95% 1,04; 1,56) (Tabela 4).

## Discussão

A conclusão da qualificação profissional a distância para o manejo da obesidade no SUS foi associada a ser nutricionista e ter acesso prévio a materiais impressos/digitais sobre o tema. Isso indicou que a área de atuação e dispor de materiais para apoiar a realização da qualificação profissional a distância influenciam diretamente o engajamento, a permanência e a conclusão de processos formativos.

Algumas limitações devem ser consideradas. A ausência de coleta de dados individuais no momento da inscrição resultou em perdas importantes de observações, o que indicou a necessidade de investigar fatores associados ao primeiro acesso e ao engajamento inicial em cursos de qualificação. Apesar da prioridade de inscrição dos profissionais e graduandos das macrorregiões Norte, Nordeste e Centro-Oeste, houve predomínio de participantes do Sul e do Sudeste, o que sugeriu a necessidade de adoção de estratégias adicionais para ampliar o acesso equitativo a cursos de qualificação no país, como divulgação direcionada, suporte tecnológico e incentivos institucionais.

Não foi possível realizar a análise de regressão multinível conforme proposto nos objetivos deste artigo, o que limitou a compreensão detalhada da influência simultânea dos fatores individuais e contextuais na conclusão do curso. Entretanto, isso pode significar que, para concluir cursos de qualificação a distância, os fatores individuais ou mesmo os fatores contextuais do micro contexto, como aqueles relacionados ao ambiente das unidades de saúde, podem ser mais importantes. Nesse sentido, sugere-se que outros estudos sejam realizados, visando identificar o papel do macro e do micro contexto na qualificação profissional no âmbito do SUS.

Ainda que os fatores contextuais não possam ser analisados em conjunto com os individuais, seus resultados podem fornecer informações úteis para os gestores de saúde. Uma maior proporção de alunos concluintes atuava em municípios com maior densidade populacional e despesas com saúde. Essas diferenças podem ser explicadas pelo fato de a maior parte dos participantes concluintes atuar na macrorregião Sudeste e em regiões metropolitanas. Panorama semelhante foi verificado em cursos autoinstrucionais da Universidade Aberta do Sistema Único de Saúde ^20^ e na qualificação profissional voltada para ações coletivas de promoção da alimentação adequada e saudável ^11^.

As UF com maiores densidades populacionais e melhores infraestruturas tendem a oferecer melhores condições de acesso à internet, o que pode impactar positivamente a participação e a conclusão em cursos a distância ^21^. No Brasil, existem graves disparidades na disponibilização de redes de internet, com maior densidade de infraestrutura de comunicação nas macrorregiões Sudeste e Sul e menor nas macrorregiões Norte e Nordeste ^22^. Esse cenário reforça a necessidade de políticas públicas que garantam equidade no acesso de cursos de qualificação da educação a distância em todo o país, incluindo maior inclusão digital em todas as macrorregiões.

Entre os fatores associados à conclusão do curso, ser nutricionista foi importante. A proximidade do tema obesidade com a área de nutrição pode ter contribuído para esses resultados. Entretanto, devido à natureza multifatorial da obesidade, esse é um tema multiprofissional e intersetorial ^7^^,^^23^, que demanda a participação de diferentes categorias profissionais da área da saúde, sobretudo médicos, enfermeiros, nutricionistas, psicólogos e profissionais de educação física. Para que isso seja possível, é imprescindível fornecer incentivo institucional e infraestrutura adequada, incluindo carga horária protegida para realização de educação permanente, acesso de qualidade aos recursos tecnológicos e reconhecimento da qualificação na progressão na carreira.

Diferentes categorias profissionais precisam se qualificar para enfrentar os desafios do manejo da obesidade, especialmente na condução de grupos terapêuticos. Estudo exploratório quantitativo, baseado na web, realizado em 2018 com 1.323 profissionais de saúde brasileiros, mostrou que 72% dos profissionais concordavam com a afirmação que “É muito difícil tratar indivíduos com obesidade”. Mais da metade dos entrevistados confirmou que a falta de qualificação/treinamento repercutia negativamente no bom desempenho das ações (75%) e que o tempo para planejar ações coletivas era insuficiente (61%). Para os enfermeiros, o atendimento em grupo de indivíduos com obesidade era a abordagem mais difícil de ser realizada (89%), sendo esse valor de 49% para os nutricionistas ^8^.

Além da categoria profissional, alunos que tiveram acesso prévio a materiais impressos/digitais sobre o manejo da obesidade apresentaram maiores chances de concluir o curso. Esse resultado reforçou a importância de disponibilizar recursos educacionais e estimular continuamente a educação permanente no serviço, visando alcançar o engajamento e a continuidade do aprendizado ^24^. O aumento progressivo da prevalência de obesidade na população adulta brasileira ^4^ e, consequentemente, o não alcance da meta de deter o seu crescimento até 2022 ^25^, reforça a urgência de expandir a visibilidade e a oferta de materiais instrucionais, sobretudo impressos devido às limitações de acesso digital nas unidades de saúde. A ampla oferta de qualificações profissionais, de forma a atingir toda a Rede de Atenção à Saúde, é necessária, visando promover o adequado enfrentamento desse desafio de saúde pública.

A escassez de materiais instrucionais disponíveis aos profissionais de saúde pode estar ligada à fragilidade na articulação entre educação permanente, vigilância e atenção à saúde, que deveriam trabalhar juntas na produção e na difusão desses recursos. No Brasil, apesar da crescente priorização política do tema da obesidade ^7^^,^^20^^,^^26^^,^^27^^,^^28^, a disponibilização efetiva de materiais de apoio nos serviços de saúde e o acesso a cursos de qualificação ainda são frágeis. Entre esses desafios, destacam-se a escassez de recursos para imprimir e distribuir os materiais impressos e a falta da devida visibilidade e divulgação dos materiais digitais e sua utilização no ambiente das unidades de saúde devido às limitações de conectividade, que também dificultam a realização de cursos a distância ^8^.

Entre as potencialidades deste estudo, destacou-se a sua temática ao abordar os fatores associados à conclusão de curso de qualificação profissional a distância para o manejo da obesidade no SUS, contemplando todas as macrorregiões e UF do país. Seus resultados possuem potencial para subsidiar o aprimoramento de futuras ofertas de qualificação profissional realizadas pelo Ministério da Saúde, bem como para orientar estratégias governamentais voltadas para o fortalecimento da educação permanente em saúde, visando à adequada formação dos profissionais de saúde do SUS e à ampliação da capacidade da Rede de Atenção à Saúde para lidar com a epidemia da obesidade no país.

Apesar da participação em cursos de qualificação profissional ser central para o aprimoramento contínuo do cuidado longitudinal dos usuários do SUS, há desafios persistentes, como o incentivo insuficiente da gestão e a consequente desmotivação dos profissionais, e a sobrecarga de trabalho, que dificultam a participação e a conclusão dos cursos ^11^^,^^29^. Isso pôde ser verificado neste estudo, ao mostrar que cerca de 60% dos participantes que se inscreveram não chegaram a ingressar na plataforma Moodle para iniciar o curso.

Para mudar esse cenário, é fundamental o apoio dos gestores para fomentar o engajamento, assegurando horários protegidos para o estudo, infraestrutura adequada e reconhecimento formal da participação em cursos de qualificação. A estabilidade empregatícia pode facilitar a busca por atualização profissional. Profissionais concursados com Planos de Carreira, Cargos e Salários do SUS que preveem a educação permanente como critério para progressão na carreira ^30^ podem ter maior interesse e capacidade de finalizar cursos de qualificação. Isso pode ser visto neste estudo, ao verificar que a maioria dos alunos concluintes era composta por profissionais concursados e com maior tempo de atuação no SUS. Além disso, é necessária a adoção de estratégias que promovam a equidade entre as UF e as macrorregiões do país.

Os resultados deste estudo apontaram que ser nutricionista e ter acesso prévio a materiais impressos/digitais aumentavam as chances de conclusão da atividade de qualificação profissional para o manejo da obesidade. Esses resultados revelaram a necessidade de ampliar o incentivo à participação de outras categorias profissionais em atividades de qualificação profissional sobre a temática, incluindo a disseminação prévia de materiais sobre o tema, visando potencializar os impactos da educação permanente na prática profissional e na qualidade do cuidado ofertado no SUS para indivíduos com obesidade.

## Data Availability

O conteúdo do banco de dados está disponível mediante solicitação ao autor correspondente.
